# A Novel Multivariate Cutting Force-Based Tool Wear Monitoring Method Using One-Dimensional Convolutional Neural Network

**DOI:** 10.3390/s22218343

**Published:** 2022-10-30

**Authors:** Xu Yang, Rui Yuan, Yong Lv, Li Li, Hao Song

**Affiliations:** 1 Key Laboratory of Metallurgical Equipment and Control Technology, Ministry of Education, Wuhan University of Science and Technology, Wuhan 430081, China; 2 Hubei Key Laboratory of Mechanical Transmission and Manufacturing Engineering, Wuhan University of Science and Technology, Wuhan 430081, China

**Keywords:** tool wear condition monitoring, multivariate cutting force signals, modified multiscale permutation entropy, one-dimensional convolutional neural network

## Abstract

Tool wear condition monitoring during the machining process is one of the most important considerations in precision manufacturing. Cutting force is one of the signals that has been widely used for tool wear condition monitoring, which contains the dynamical information of tool wear conditions. This paper proposes a novel multivariate cutting force-based tool wear monitoring method using one-dimensional convolutional neural network (1D CNN). Firstly, multivariate variational mode decomposition (MVMD) is used to process the multivariate cutting force signals. The multivariate band-limited intrinsic mode functions (BLIMFs) are obtained, which contain a large number of nonlinear and nonstationary tool wear characteristics. Afterwards, the proposed modified multiscale permutation entropy (MMPE) is used to measure the complexity of multivariate BLIMFs. The entropy values on multiple scales are calculated as condition indicators in tool wear condition monitoring. Finally, the one-dimensional feature vectors are constructed and employed as the input of 1D CNN to achieve accurate and stable tool wear condition monitoring. The results of the research in this paper demonstrate that the proposed approach has broad prospects in tool wear condition monitoring.

## 1. Introduction

As an important part of high-speed milling machines, the tool experiences intense friction with the workpiece and the chip during the machining process. Under the continuous change of thermodynamic coupling, it will inevitably produce wear until the tool fails. If the failed tool is not replaced in time, the machining accuracy of the workpiece will decrease, and in serious cases, the workpiece will be scrapped, and the machine tool will fail, seriously affecting the machining efficiency. Therefore, it is of great significance to realize the in situ tool wear condition monitoring during the automated manufacturing process, which can guarantee the stability of machine processing quality and improve production efficiency [1].

There are two methods to realize tool wear condition monitoring: direct and indirect measurement methods. Most direct measurement methods rely on visual, optical or image measurement methods to directly measure the tool wear condition [2,3], which has the advantage of being intuitive and has high accuracy. However, the online monitoring of tool wear conditions cannot be realized because the measurement needs to stop the machining process. The indirect measurement method is used to collect relevant signals during the process of tool machining, process the signals and extract the characteristics that can characterize the tool wear and then monitor the tool wear condition using machine learning. Commonly used monitoring signals include cutting force signals [4,5], vibration signals [6,7] and current signals [8,9], etc. With the rapid development of signal processing and pattern recognition, the indirect measurement method has gradually become the mainstream method to identify tool wear conditions.

The original signals collected by sensors contain noise and other components, and the nonlinear characteristics of tool wear are often hidden in the original signals, so it is necessary to use signal processing methods to process the data. Commonly used signal analysis methods, such as wavelet analysis [10,11], empirical mode decomposition (EMD) [12,13] and variational mode decomposition (VMD) [14,15], have been effectively applied in tool wear condition monitoring. Wavelet analysis is suitable for extracting wear features from nonlinear and nonstationary tool wear signals. However, the selection of an appropriate basis function requires some engineering experience, which will affect the extraction effect of tool wear characteristics. EMD [16] is an adaptive nonlinear nonstationary signal processing method which has no requirements for basic functions. However, EMD has problems with mode aliasing and end effects [17]. VMD [18] introduces a variational model to transform the signal decomposition into finding the optimal solution of the constraint model so as to avoid the mode aliasing phenomenon caused by local mutation and has a certain advantage in computing speed. However, VMD has the limitation of not being able to synchronously process multivariate signals collected by a multi-sensor acquisition system. Compared with single-channel signals, multi-channel signals can comprehensively collect dynamic information and effectively eliminate information uncertainty [19,20]. The cross information of multi-channel signals can improve the reliability of the acquired information, so the tool wear monitoring methods based on multi-channel signals have been widely concerned. Multivariate variational mode decomposition (MVMD) [21] inherits the advantages of VMD and expands it from a single channel to multiple channels. MVMD can decompose multiple signals into multiple sets of band-limited intrinsic mode functions (BLIMFs), and the same frequency characteristics can occur in the same order BLIMFs, avoiding the problem of frequency misalignment. MVMD is able to obtain more accurate BLIMFs, which can effectively alleviate the mode aliasing problem. Based on the above advantages, MVMD can provide a new way to process multivariate signals [22].

Entropy is a method which can effectively measure the complexity of time series. Commonly used entropies include permutation entropy (PE) [23], sample entropy (SE) [24] and fuzzy entropy (FE) [25]. Bandt et al. [26] proposed permutation entropy based on the idea of comparing adjacent values to measure the complexity of the time series. Aziz et al. [27] proposed a multiscale permutation entropy (MPE) algorithm through a coarse graining process. Compared with PE, MPE can extract information at multiple scales in time series and more comprehensively represent the complexity of the mechanical system [28], which has been widely concerned. However, the length of the time series will be reduced after the coarse graining process, which affects the calculation accuracy and stability of the entropy value. To improve the stability and accuracy of the traditional MPE, modified multiscale permutation entropy (MMPE) was proposed by the authors of this paper [29]. MMPE improves the traditional coarse graining process and effectively solves the mutation of entropy when dealing with short time series so that the permutation entropy with good stability and high precision can be obtained. The superiority of MMPE in nonlinear characteristic extraction has been proved. Therefore, it can be applied in tool wear monitoring as a wear feature extraction method.

Deep learning has been highly successful in many fields, such as computer vision and speech recognition, of which the convolutional neural network (CNN) is the most widely applied. Compared with traditional machine learning models [30], the advantages of CNN are the combination of feature extraction and classification and the ability to perform adaptive screening of features. Therefore, CNN has been widely used in tool wear condition monitoring [31,32]. However, two-dimensional CNN (2D CNN) is more frequently used. Compared with 2D CNN, the biggest difference between 1D CNN and 2D CNN is in the size of the convolution kernel. The following are some advantages of 1D CNN over 2D CNN: (1) one-dimensional convolution only needs vector calculation, without complex matrix calculation, which greatly improves the calculation efficiency. (2) 1D CNN has fewer training parameters and can perform pattern recognition with fewer samples and epochs [33]. Based on the above advantages, 1D CNNs have been widely used in structural health monitoring [34,35]. In recent years, more and more scholars have begun to apply 1D CNN to the field of tool wear monitoring. Kuo et al. [36] established 1D CNN model similar to the DenseNet framework. By adjusting the frame and parameters of the model, high precision prediction of cutting tool wear was achieved. Xu et al. [37] proposed a new tool wear monitoring method based on deep learning. 1D CNN model with residual blocks and dilated convolution was developed to monitor and predict tap tool wear. Therefore, 1D CNN can provide a new reference for tool wear condition recognition.

A novel multivariate cutting force-based tool wear monitoring method using one-dimensional convolutional neural network is proposed. MVMD is used to decompose multivariate cutting force signals into multiple sets of multivariate BLIMFs. MMPE values at multiple time scales are calculated and used to measure the complexity of multivariate BLIMFs. One-dimensional feature vectors are constructed and employed as the input of 1D CNN to achieve in situ tool wear condition monitoring. The novelty and the contributions of the proposed method can be summarized as follows:

(1) The multiscale entropy analysis can provide better support for deep learning. The MMPE analysis method based on BLIMFs can accurately extract the dynamical properties of multivariate cutting force signals. 1D CNN is employed to screen wear features and identify wear conditions. This strategy can reduce the training parameters of 1D CNN while ensuring tool wear monitoring accuracy.

(2) The proposed method can fuse the wear feature information of multi-channel signals into a one-dimensional vector. The method of multi-channel feature fusion can describe the change of tool wear more comprehensively. Comparative experiments show the superiority of the proposed method. Case studies indicate that the proposed method can be promising.

Section 2 reviews the methodologies of MVMD, MMPE and 1D CNN. In Section 3, a novel multivariate cutting force-based tool wear monitoring method using one-dimensional convolutional neural network is proposed. Section 4 verifies the proposed method through the analysis of experimental data sets and results as well as comparative experiments. Section 5 summarizes the whole paper and introduces the main research results of this paper.

## 2. Methodologies

### 2.1. Multivariate Variational Mode Decomposition

MVMD is an adaptive decomposition algorithm which can decompose multi-channel cutting force signals cooperatively. As an extension of the original VMD algorithm, the main purpose of MVMD is to extract multivariable modulated oscillation signals from the original multi-channel signals [21].

If the preset number of multivariable modulated oscillation signals is K, then:

(1) $$x\left(t\right)=\sum_{k=1}^{K}u_{k}(t)$$

Multivariable oscillation modes $\left\{u_{k}\left(t\right)\right\}$ should be extracted from the original signal to minimize the sum of the bandwidths of the extracted modes. At the same time, $\left\{u_{k}\left(t\right)\right\}$ can effectively restore the original signal x(t). Therefore, the bandwidth of $u_{k}\left(t\right)$ is estimated using the square $L^{2}$ norm of the demodulation signal $u_{k}^{+}\left(t\right)$ gradient.

(2) $$g=\sum_{k=1}^{K}{\left\|\partial_{t}(e^{-j\omega_{k}t}u_{k}^{+}(t))\right\|}_{2}^{2}$$

It is necessary to estimate the bandwidth of the modulated multivariable oscillation signal to determine the multi-component oscillation with a single common frequency component $\omega_{k}$ in multi-channel. The single side spectrum of each channel in $u_{k}^{+}\left(t\right)$ is offset by the central frequency $\omega_{k}$, and the Frobenius norm is used to convert the single channel into a multi-channel signal.

The Frobenius norm of matrix W is defined as the square root of the sum of absolute values of the elements $w_{ij}$ of matrix W, namely

(3) $${\left\|W\right\|}_{F}=\sqrt{\sum_{i}\sum_{j}w_{i,j}^{2}}$$

Then, it is reflected in the MVMD algorithm,

(4) $$g^{\prime }=\sum_{k}\sum_{h}{\left\|\partial_{t}(e^{-j\omega_{k}t}u_{k,n}^{+}(t))\right\|}_{2}^{2}$$

where $u_{k,n}^{+}\left(t\right)$ is the analytical signal with the number of channels n and models k. A variational constraint model is constructed, namely

(5) $$\begin{array}{l} \underset{\left\{u_{k,n}\},\{\omega_{k}\right\}}{\min}\{\sum_{k}\sum_{n}{\left\|\partial_{t}(e^{-j\omega_{k}t}u_{k,n}^{+}(t))\right\|}_{2}^{2}\}\\ s.t.X_{N}(t)=\sum_{k}u_{k,n}^{+}(t),n=1,2,\cdots ,N \end{array}$$

According to Formula (5), if multiple linear constraints exist, the corresponding augmented Lagrange function can be obtained.

(6) $$\begin{array}{l} L(\{u_{k,n}\},\{\omega_{k}\},\lambda_{n})=\alpha \sum_{k=1}^{K}\sum_{h=1}^{N}{\left\|\partial_{t}(e^{-j\omega_{k}t}u_{k,n}^{+}(t))\right\|}_{2}^{2}\\ +\sum_{n=1}^{N}{\left\|x_{n}(t)-\sum_{k=1}^{K}u_{k,n}(t)\right\|}_{2}^{2}+\sum_{n=1}^{N}\langle \lambda_{n}(t),x_{n}(t)-\sum_{k=1}^{K}u_{k,n}(t)\rangle \end{array}$$

Using the alternating direction multiplier method to update $u_{k}$, $\omega_{k}$ and Lagrange operator λ, the optimal solution of the variational model can be obtained.

(7) $${\hat{u}}_{k,n}^{l+1}(\omega )=\frac{{\hat{x}}_{n}(\omega )-\sum_{i-1}^{k=1}{\hat{u}}_{i,n}^{l+1}(\omega )+\frac{{\hat{\lambda }}^{l}(\omega )}{2}}{1+2\alpha {\left(\omega -\omega_{k}^{l}\right)}^{2}}$$

where α is the penalty factor and l is the number of iterations. Since the last two terms of the augmented Lagrange function do not depend on $\omega_{k}$, the related problem can be simplified as

(8) $$\omega_{k}^{n+1}=\underset{\omega_{k}}{\arg\min}\left\{\sum_{n}{\left\|\partial_{t}(e^{-j\omega_{k}t}u_{k,n}^{+}(t))\right\|}_{2}^{2}\right\}$$

Let the first derivative of the above quadratic function be 0 to minimize the sum of the quadratic functions, then

(9) $$\omega_{k}^{l+1}=\frac{\sum \int_{0}^{\infty }\omega {\left|{\hat{u}}_{k,n}^{l+1}(\omega )\right|}^{2}d\omega }{\sum \int_{0}^{\infty }{\left|{\hat{u}}_{k,n}^{l+1}(\omega )\right|}^{2}d\omega }$$

### 2.2. The Proposed Modified Multiscale Permutation Entropy

MPE combines the coarse graining process with the PE calculation process. By calculating the entropy value of the coarse-grained time series, the calculation results can be obtained at different time scales. MPE can extract more dynamic feature information from the time series and comprehensively describe the changes in the mechanical system, so more condition indicators can be obtained compared with the original single scale. The MPE algorithm consists of two main steps [27], which are detailed as follows.

(1) The time series $X=\left\{x_{i},i=1,2,\cdots ,N\right\}$ with sequence length N are segmented by the coarse graining process.

(10) yjτ=1τ∑i=(j−1)τ+1jτxi, j=1,2,⋯,[Nτ]

where $\left[\frac{N}{\tau }\right]$ indicates the round of $\frac{N}{\tau }$; τ is the scale factor.

(2) By calculating PE values of coarse-grained time series, the MPE can be obtained.

(11) $$\mathrm{MPE}(x,\tau ,m,\delta )=\mathrm{PE}(y_{j}^{\tau },m,\delta )$$

where m and δ are the embedded dimension and delay parameter, respectively.

Although MPE can represent characteristic information in time series, it still has shortcomings. ① In the process of coarsening, the processing of each data point is not completely consistent, which may lead to the mutation of the PE value. ② When calculating PE, the more points the time series has, the more stable the entropy value will be. However, multi-scale analysis will reduce the length of the time series. With the increase in scale factor, the signal length used to calculate the entropy value will be shorter, which will lead to the increase in entropy error. To improve the deficiency of MPE, the traditional coarse graining process must be improved. At the same scale factor τ, MMPE can obtain τ time series after coarse graining treatment and then calculate its average value [29]. The steps of this method are as follows:

(1) For a given time series processed with an improved coarse graining process, τ new time series can be obtained.

(12) $$y_{i,j}^{\tau }=\frac{\sum_{f=0}^{\tau -1}x_{f+i+\tau (j-1)}}{\tau }$$

(13) $$z_{i}^{\tau }=\left\{y_{i,1}^{\tau },y_{i,2}^{\tau },\cdots \right\},\;i=1,2,\cdots ,\tau$$

In calculating MPE, one scale factor τ corresponds to one coarse-graining sequence $y_{j}^{\tau }$, while in calculating MMPE, one scale factor τ corresponds to τ coarse-graining sequence $z_{i}^{\tau }$.

(2) PE of each coarse-grained time series corresponding to the scale factor τ can be calculated, respectively, and then its average value is obtained. Finally, MMPE can be obtained.

(14) $$\mathrm{MMSE}(x,\tau ,m,\delta )=\frac{1}{\tau }\sum_{i=1}^{\tau }\mathrm{PE}(z_{i}^{\tau })$$

### 2.3. One-Dimensional Convolutional Neural Network

**Figure 1:**
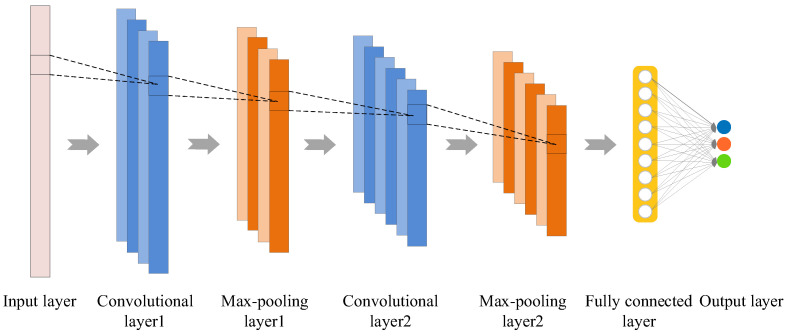
The structure of 1D CNN model.

1D CNN is a typical neural network employing one-dimensional convolution for feature extraction from one-dimensional time series. Similar to 2D CNN, it also includes parts such as convolutional layer, pooling layer, fully connected layer and output layer. 1D CNN is able to automatically extract features from time series layer by layer using alternating arrangements of convolutional and pooling layers. The extracted features are fed to the fully connected layer and the output layer for state identification. Therefore, 1D CNN can fuse feature extraction and classification [33]. Figure 1 illustrates the structure of 1D CNN model.

(1) Convolutional Layer

In the convolutional layer, the input feature matrix is locally convolved with the preset convolution kernels according to the step size. After traversing the convolution operation on the input feature matrix, the corresponding feature matrix is output. The convolution kernel of 1D CNN is one-dimensional [38], and the one-dimensional convolution operation can be represented as:

(15) $$x_{k}^{l}=\sum_{i=1}^{N_{l-1}}conv1D(w_{ik}^{l-1},s_{i}^{l-1})+b_{k}^{l}$$

where $x_{k}^{l}$ and $b_{k}^{l}$ are the input and bias of the kth neuron in the *l*th layer, respectively; $w_{ik}^{l-1}$ is the convolution kernel between the ith neuron in the l−1 layer and the kth neuron in the lth layer; $s_{i}^{l-1}$ is the output of the ith neuron in the l−1 layer; $N_{l-1}$ is the number of neurons in the l−1 layer; conv1D(∗) is the one-dimensional convolution operation.

(2) Pooling Layer

The main purpose of the pooling layer is to reduce network parameters and computation by changing the width of the feature matrix while preserving important features. The pooling operation can be mainly divided into the average pooling operation and the maximum pooling operation. In this paper, the maximum pooling operation is used. The maximum value in the adjacent area of a certain location is taken as the final output of this location.

(16) $$s_{k}^{l}=\underset{(k-1)H+1\leq j\leq kH}{\max}(s_{j}^{l-1})$$

where H is the width of the convolution kernel.

(3) Batch Normalization

To avoid gradient dispersion of neurons at each layer and accelerate the speed of network convergence, batch normalization can be used to standardize each hidden layer so that each layer has the same Gaussian distribution, which has a mean of 0 and variance of 1.

(17) $$x_{i}^{\prime }=\frac{x_{i}-\mu_{B}}{\sqrt{\sigma_{B}^{2}+\varepsilon }},\;y_{i}=\gamma x_{i}^{\prime }+\beta$$

where $\mu_{B}$ is the mean of batch date, $\sigma_{B}$ is the standard deviation of batch data, ε is to avoid infinite decimals with a zero denominator and $x_{i}$ and $x_{i}^{\prime }$ are the input before and the intermediate output of the batch normalization of the ith neuron, respectively. γ and β are the scale change factor and offset factor introduced to restore network expression ability, respectively, which are obtained by network learning, and $y_{i}$ is the final output of neurons after batch normalization.

## 3. A Novel Multivariate Cutting Force-Based Tool Wear Monitoring Method Using One-Dimensional Convolutional Neural Network Is Proposed

During the machining process, the milling cutter removes excess material from the workpiece according to the preset machining parameters. The milling cutter causes severe friction with the workpiece and chips. These cause the surface of the milling cutter to be slowly eliminated, resulting in tool wear. When tool wear occurs, the contact between the tool and the workpiece will change, which will lead to a change in friction, resulting in a corresponding change in cutting force. Therefore, cutting force can directly reflect the change in tool wear condition during the machining process. The studies on IMFs and BLIMFs show that this algorithm can accurately describe the dynamic characteristics of signals [39,40]. The MMPE algorithm was proposed in the previous research [29] by one of the authors of this paper. MMPE is not only able to accurately characterize the complexity of dynamic systems but also to suppress sudden changes in entropy, which is suitable for tool wear monitoring. Moreover, according to the author’s previous studies, the MMPE algorithm has superior transform resistance to noise. When processing signals with different SNRs, MMPE values have no mutation in most time scales and have good stability. Therefore, the proposed MMPE analysis method based on BLIMFs can not only quantitatively measure the complexity of dynamic systems but also has certain noise resistance.

A novel multivariate cutting force-based tool wear monitoring method using one-dimensional convolutional neural network is proposed. The MMPE analysis method based on BLIMFs is used to extract wear characteristics from multivariate cutting force signals, and wear condition identification is carried out through 1D CNN to realize in situ monitoring of tool wear. The main algorithms of the proposed approach are presented as follows:

(1) MVMD is used to process multivariate cutting force signals, and the multiple sets of multivariate BLIMFs containing a large number of nonlinear and nonstationary wear characteristics are obtained.

(2) Afterwards, the values of the proposed MMPE at multiple time scales are calculated and used as condition indicators to measure the complexity of multivariate BLIMFs. MMPE can accurately characterize the complexity of the dynamic system and suppress the sudden change of entropy, which is suitable for tool wear monitoring.

(3) One-dimensional feature vectors are constructed by realigning MMPE values of multivariate BLIMFs into one column vector. The feature vectors denote the dynamical properties of tool wear conditions. All feature vectors are randomly divided into training set, validation set and testing set.

(4) The training set and validation set are input into 1D CNN for training, and the parameters are adjusted during the training process to obtain a neural network model with good recognition. The trained 1D CNN model is used to test the testing set to realize in situ monitoring of tool wear conditions.

## 4. Application Research by Experimental Data Analysis

### 4.1. Experimental Data Description

During the machining process, when the cutting edge of the tool cuts into the workpiece, there will be intense friction between the two, which causes the temperature in the contact area to rise sharply. Due to the effect of high temperature, high pressure and constant physical impact, various physical and chemical reactions occur in the contact area, resulting in constant wear of the tool. When the tool wear continues to a certain degree, the cutting force will increase, the cutting temperature will increase and the cutting performance of the tool will rapidly decline. The nonlinear and complex wear mechanism of the tool is difficult to be described by a complete and accurate mathematical mechanical model. The goal of our research is to achieve in-site tool wear monitoring in terms of signal processing.

**Figure 2:**
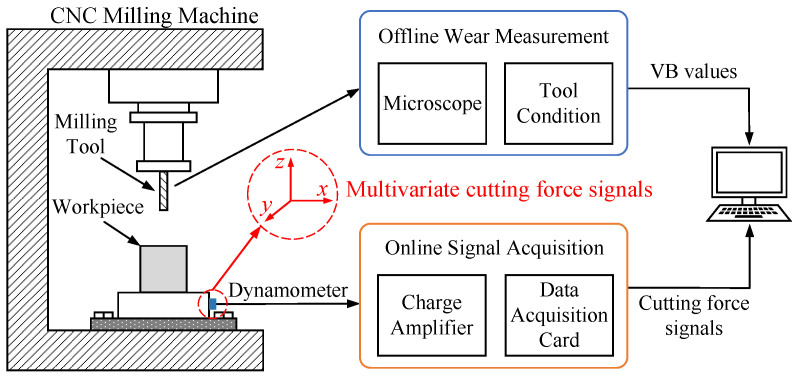
High-speed milling machine tool wear monitoring experimental platform.

Experimental data were obtained from the 2010 Prognostic and Health Management Society Conference Data Challenge [41]. This paper follows the international standard ISO in which the VB amount measured on the flank face at half the depth of cut is used as the actual wear of the milling tool. The milling experiment was carried out on a high-speed CNC milling machine, and the workpiece was cut by dry milling. A three-edged ball-end tungsten carbide tool was selected as the dry milling tool, and the cutting material was a square stainless steel workpiece with hardness of HRC52. The machining conditions for the milling process were a spindle speed of 10,400 r/min, feed rate of 1555 mm/min, axial depth of cut of 0.2 mm and radial depth of cut of 0.125 mm [42]. During the milling process, the triaxial cutting force signals in the form of electric charge were collected by the sensor and sent to the data acquisition card through the charge amplifier. The data acquisition card converted electrical signals into digital signals and stored them in the computer. The experimental platform used in this milling experiment is shown in Figure 2. The specific hardware equipment and milling conditions are shown in Table 1.

**Figure 3:**
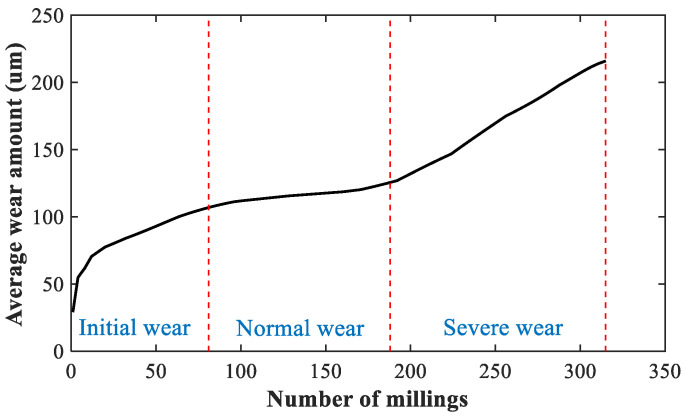
The change curve of the milling tool average wear in the sixth experiment [43].

Six life cycle experiments were carried out under the above milling conditions, and the experimental data of six milling tools were obtained. During the milling experiment, multivariate cutting force signals in the X, Y and Z axes were collected. Since the milling workpiece was square and each cutting length was 108 mm, each cutting cycle time was equal. At the end of each cutting cycle, the machine was stopped, the milling tool was taken off and the microscope was used to measure the flank wear of the milling tool’s three cutting edges, which were recorded as flute_1_, flute_2_ and flute_3_. The collected and measured data were saved as an experimental sample, and the experiment was stopped when the milling tool was severely worn and could not work. A total of 315 samples were obtained [43]. In this paper, multivariate cutting force signals in the X, Y and Z axes of the sixth experiment were selected as the research object, and the average wear values of the three cutting edges of the milling tool were taken as the actual wear of the milling tool. Figure 3 is the average wear values curve of the milling tool. According to the changes in milling tool wear, the cutting tool wear conditions in the sixth experiment were divided into three different conditions, as shown in Table 2.

### 4.2. Quantitative Feature Extraction Based on Multivariate Cutting Force Signals

**Figure 4:**
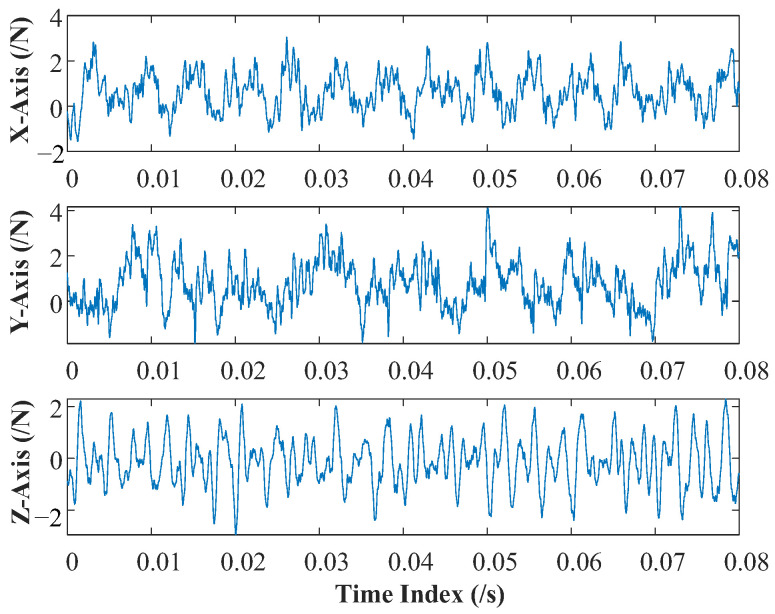
Time domain diagram of multivariate cutting force signals.

To avoid the influence of the beginning and end of cutting on the milling tool wear condition, a total of 4096 data points from 50,001 to 54,096 in each cutting cycle were selected as sample data in this paper. Multivariate cutting force signals under different wear conditions hide different nonlinear and nonstationary wear characteristics. Because of the complex frequency components of multivariate cutting force signals, it is not conducive to feature extraction directly, so the signals need to be processed in advance. Taking a group of multivariate cutting force signals at the initial wear condition as an example, the time domain diagram is shown in Figure 4.

**Figure 5:**
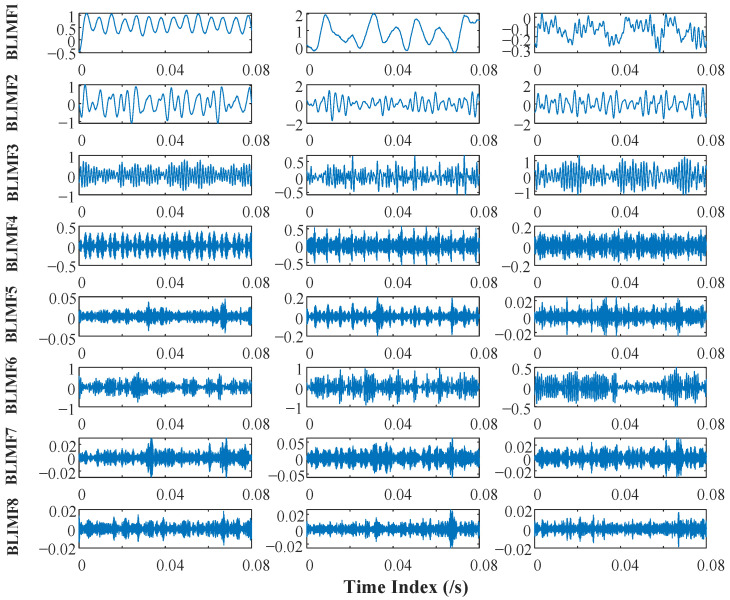
The time spectrum of the first 8 orders BLIMFs generated by MVMD.

Using MVMD to decompose the multivariate cutting force signals, multiple sets of multivariate BLIMFs containing a large number of nonlinear and nonstationary wear characteristics can be obtained. After comparing the test results many times, the decomposition parameter K=8, the penalty factor α=2000 and the default values of other parameters were selected. The decomposition results of multivariate cutting force signals are shown in Figure 5.

MVMD can decompose multivariate cutting force signals with complex frequency components into multiple sets of BLIMFs with simple frequency components. BLIMFs of different orders are used to represent different frequency characteristic components of the original signals, which fully describes the dynamic information of original signals. Therefore, multivariate BLIMFs contain a lot of nonlinear and nonstationary wear features and are more conducive to the feature extraction of tool wear compared with original signals. After the decomposition of multivariate signals, MMPE is used to extract the quantitative features. Three parameters in MMPE need to be preset before calculation: embedding dimension m, delay parameter δ and scale factor τ. The value range of embedding dimension m is generally 3≤m≤7. When m≤2, the effectiveness of the algorithm will be reduced due to the small amount of important information contained in the reconstructed time series. However, if the m value is too high, the amount of calculation will increase, and it is difficult to describe the subtle change in time series [44]. Therefore, m=4 was determined in our research. Since the delay parameter δ had little effect on the final calculated result, δ was preset as 1. Scale factor τ determines the degree of coarsening. According to the research needs, scale factor τ was preset as 20.

**Figure 6:**
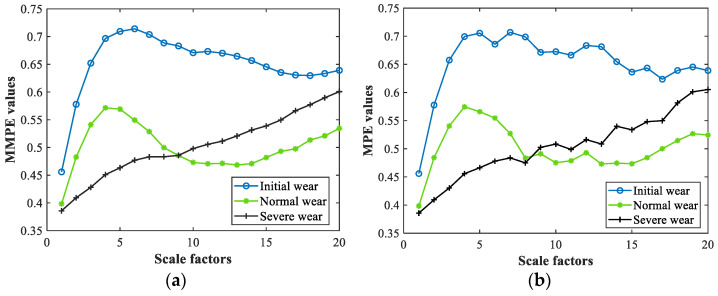
Entropy values denoting 3 different wear conditions. (**a**) MMPE and (**b**) MPE values.

The multivariate cutting force signals under three different wear conditions were decomposed by MVMD, and MMPE values under 20 scale factors of the first eight orders BLIMFs were calculated. Taking the MMPE values of the BLIMF of the same order and the same channel under different wear conditions as examples, the MMPE values change curve was drawn. To compare with MPE, the same signals were processed in the same way, the MPE values were obtained and the curve was drawn. The entropy values curves of MMPE and MPE are illustrated in Figure 6a,b, respectively.

It can be seen from Figure 6 that the MMPE and MPE curves of the same BLIMF show roughly the same change trend under different scale factors. However, the MPE values curve sometimes fluctuates greatly with the change of scale factors, and some MPE values have abrupt changes. The MMPE values curve is more stable than the MPE curve, and there is no obvious entropy mutation. Compared with the traditional method, the modified coarse graining process can obtain more accurate results, reduce calculation error and inhibit the occurrence of mutation. Therefore, it can be proved that MMPE has good stability. Figure 6a shows that MMPE values in different wear conditions overlap when the scale factor is 9, which indicates the limitations of entropy values in characterizing tool wear conditions at a single scale. MMPE values at multiple scales can better obtain the characteristic information of tool wear, reflecting the complexity of multivariate cutting force signals under different wear conditions. To improve the computational efficiency and recognition rate, the MMPE values of the first eight scales were selected as condition indicators to construct one-dimensional feature vectors after several experiments.

### 4.3. Tool Wear Condition Monitoring by One-Dimensional Convolutional Neural Network

The multivariate cutting force signals of all 315 cutting cycles in the sixth experiment were selected for analysis in this paper. The characteristics of multivariate cutting force signals were extracted based on the above method, and one-dimensional MMPE value vectors were constructed as condition indicators. According to the change in average wear values, the sixth experiment was divided into three different wear conditions. For each wear condition, the training set, validation set and testing set were randomly divided in the ratio of 0.6:0.2:0.2. The structure of 1D CNN directly affects the efficiency and accuracy of the neural network model. The deeper the 1D CNN model structure is, the stronger the learning ability of the neural network model will be. However, the calculation time of the model will be longer, and the over-fitting phenomenon easily occurs. Therefore, the structure of 1D CNN needs to be adjusted by experience and trial. After many tests, 1D CNN model in this paper was mainly set up as two convolutional layers, two maximum pooling layers, two batch normalization layers, one flatten layer, one dropout layer and two fully connected layers.

**Figure 7:**
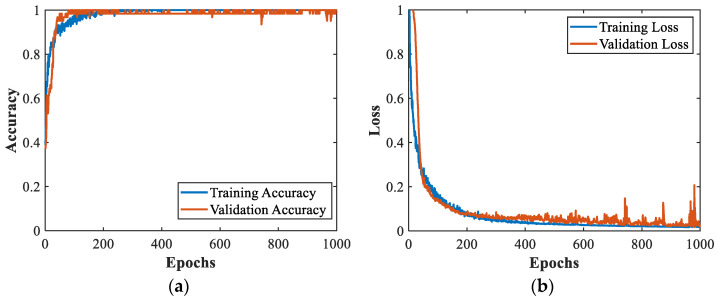
(**a**) The change curves of accuracy during 1D CNN training process. (**b**) The change curves of loss during 1D CNN training process.

The input one-dimensional MMPE value vectors were preprocessed, and features were extracted alternately through convolutional and pooling layers. The batch normalization layer was added after each convolutional layer and before the activation function to improve the efficiency of the training process and enhance the robustness of 1D CNN model [45]. After the batch normalization layer, the activation function was generally used for nonlinear transformation to improve the nonlinear expression ability of the network [46]. ReLU was used in this paper to accelerate the convergence of the model. To avoid the over-fitting phenomenon, the dropout layer was used after the fully connected layer. The output of each neuron in the hidden layer was zeroed with a probability of P=0.5 during training to reduce the interdependence between neurons [47]. Finally, wear characteristics were identified by the softmax layer. During the construction of the 1D CNN, some parameters that affect the model classification effect and training speed, such as batch size and optimizer type, need to be selected. The parameters of 1D CNN model were selected through repeated experiments and the principle of a single variable was followed in the selection process. Finally, the optimizer was Adam, the learning rate was preset as 0.0001 and the batch size was 12. In addition, the validation set also helped the neural network model to adjust the parameters during the training of 1D CNN model while being able to verify the generalization ability of the model. The training set and validation set were input into 1D CNN for training, and the parameters were adjusted during the training process to obtain a neural network model with good recognition. This paper adopted Tensorflow and TensorBoard to construct the 1D CNN model and visualize the results. The accuracy and loss change curves of 1D CNN training process are illustrated in Figure 7.

**Figure 8:**
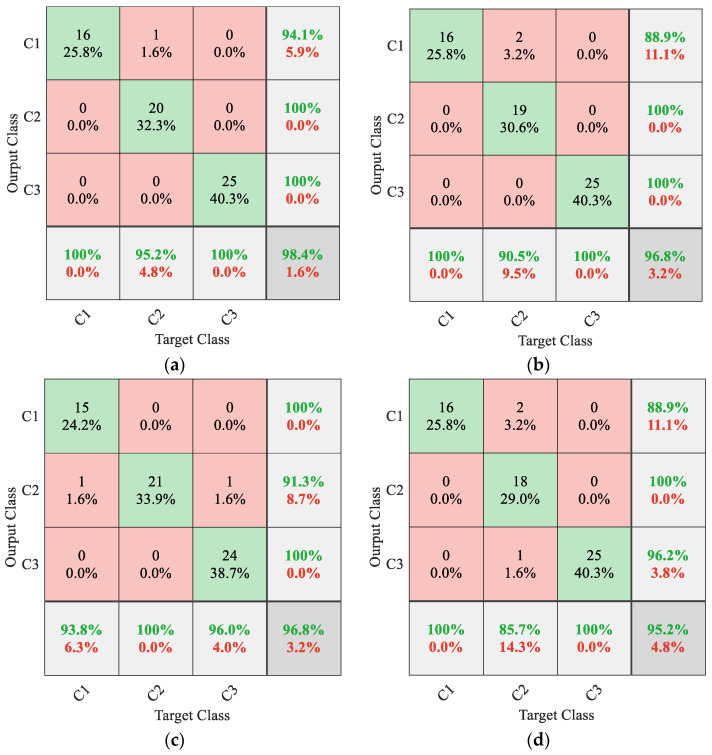
(**a**) MVMD–MMPE–1D CNN tool wear conditions monitoring result. (**b**) MVMD–MMPE–GA-SVM tool wear conditions monitoring result. (**c**) MEMD–MMPE–1D CNN tool wear conditions monitoring result. (**d**) MVMD–MPE–1D CNN tool wear conditions monitoring result.

Figure 7 shows that the accuracy of 1D CNN gradually increased during the training process. After the 200th epoch, the accuracy of the training set and validation set remained stable at a relatively high precision, and there was no large-scale oscillation phenomenon. The accuracy of the training set and validation set, respectively, were 100% and 98.39% at the end of the model training. The loss function of 1D CNN model decreased gradually with the increase in epochs and finally remained in a low range. The loss curve of the training set fit closely with that of the validation set, and there was no obvious over-fitting phenomenon during the training process. The trained 1D CNN model was used to recognize the wear condition of the testing set. To more clearly illustrate the tool wear monitoring effect of the proposed method, confusion matrix was used to visualize the results, as shown in Figure 8.

In Figure 8, the abscissa represents the real wear condition of the testing set, and the ordinate represents the predicted results of 1D CNN. Figure 8a illustrates that the prediction accuracy of 1D CNN model was 98.4%, which indicates that the proposed method can effectively extract nonlinear characteristics of tool wear and realize accurate tool wear condition classification using 1D CNN. To verify the superiority of the proposed method, several sets of different methods were compared in this paper, each of which uses the same dataset. Using the MMPE values of the first eight orders of multivariate BLIMFs obtained by MVMD as the input of the genetic algorithm-support vector machine (GA-SVM), the prediction accuracy was 96.8%, which is shown in Figure 8b. Using the MMPE values of the first eight orders of multivariate IMFs obtained by multivariate empirical mode decomposition (MEMD) as the input of 1D CNN, the prediction accuracy was 96.8%, which is shown in Figure 8c. Using the MPE values of the first eight orders of multivariate BLIMFs obtained by MVMD as the input of 1D CNN, the prediction accuracy was 95.2%, which is illustrated in Figure 8d.

Figure 8 shows that the proposed method achieved the highest monitoring accuracy. To highlight the advantages of multiscale entropy analysis, this paper compared the tool wear monitoring method using only 1D CNN. A one-dimensional vector was constructed using multivariate cutting force signals, and one-dimensional vectors were used as the input to 1D CNN to achieve tool wear condition identification. To highlight the advantages of tool wear monitoring method based on multi-channel signals, this paper also compared the methods based on single-channel cutting force signals. The cutting force signals in different axes were processed by VMD, and the MMPE values at multiple scales of BLIMFs were calculated. One-dimensional feature vectors were constructed and input to 1D CNN to achieve tool wear monitoring. To avoid the randomness of single test, the above methods were repeated 10 times in this paper, and the average accuracy of the 10 tests was taken as the evaluation standard. Finally, the average accuracy results of 10 tests with different methods are shown in Table 3.

It can be seen from Table 3 that the effectiveness of MVMD and MMPE in extracting nonlinear characteristics of tool wear and the superiority of 1D CNN in the identification of wear conditions were verified by comparative analysis of classification results of different methods. Table 3 also demonstrates the advantages of the multivariate signal-based tool wear monitoring approach and the ability of multiscale entropy analysis to provide better support for deep learning. The experimental results clearly prove the effectiveness of the multivariate cutting force-based tool wear monitoring method using 1D CNN.

## 5. Conclusions and Discussion

A novel multivariate cutting force-based tool wear monitoring method using one-dimensional convolutional neural network was proposed. Firstly, MVMD was used to process the multivariate cutting force signals, and the multiple sets of multivariate BLIMFs containing a large number of nonlinear and nonstationary wear characteristics were obtained. Afterwards, MMPE values at multiple time scales were calculated and used to measure the complexity of multivariate BLIMFs. Finally, one-dimensional feature vectors were constructed and employed as the input of 1D CNN to achieve in situ tool wear condition monitoring. The main research results of the tool wear condition monitoring method proposed in this paper are as follows:

(1) Multivariate cutting force signals were used as monitoring signals to realize tool wear monitoring in this paper. Multivariate cutting force signals contain comprehensive dynamic information on tool wear, which is suitable for extracting wear characteristics. At the same time, the research on multiple signals agrees with the rapid development trend of multi-sensor acquisition systems.
(2) MVMD and MMPE were combined to extract the characteristic information of tool wear. MVMD can decompose multivariate cutting force signals adaptively and can effectively separate the frequency components of multiple signals. MMPE can accurately characterize the nonlinear characteristics of tool wear as condition indicators.
(3) 1D CNN has strong adaptive feature extraction ability, which can reduce the error of empirical judgment and make the recognition effect more accurate and intelligent. Compared with the traditional machine learning model, 1D CNN has higher recognition ability and better monitoring effects.

To sum up, this paper provides a novel method for in situ tool wear monitoring. The superiority of the method was verified by theoretical illustration and experimental data analysis. The next research focus will be to apply the proposed method to other types of signals and extend it to other tools in the production process.

## Figures and Tables

**Table 1:** Experimental hardware and milling conditions.

Operational Parameter	Value
CNC milling machine	Roders Tech RFM760
Dynamometer	Kistler 9265B
Spindle speed	10,400 r/min
Feed rate	1555 mm/min
Z depth of cut (axial)	0.2 mm
Y depth of cut (radial)	0.125 mm
Sampling frequency	50 kHz

**Table 2:** The standard of milling tool wear condition division in the sixth experiment.

Wear Condition	Wear Label	Number of Millings
Initial wear	C1	1~81
Normal wear	C2	82~188
Severe wear	C3	189~315

**Table 3:** The average classification accuracy by different methods.

Methods	Average Classification Accuracy
VMD + MMPE + 1D CNN (X-axis)	92.91%
VMD + MMPE + 1D CNN (Z-axis)	93.23%
VMD + MMPE + 1D CNN (Y-axis)	94.03%
1D CNN	93.28%
MVMD + MPE + 1D CNN	95.48%
MEMD + MMPE + 1D CNN	96.61%
MVMD + MMPE + GA-SVM	96.77%
MVMD + MMPE + 1D CNN	97.42%

## Data Availability

Experimental data were obtained from the Prognostics and Health Management Society 2010 PHM Society Conference Data Challenge. The resource can be found in the corresponding reference.
